# Polyol-Induced 100-Fold Enhancement of Bacterial Ice Nucleation Efficiency

**DOI:** 10.1021/acs.jpcc.4c07422

**Published:** 2024-12-05

**Authors:** Galit Renzer, Rosemary J. Eufemio, Ralph Schwidetzky, Janine Fröhlich-Nowoisky, Mischa Bonn, Konrad Meister

**Affiliations:** † Department for Molecular Spectroscopy, Max Planck Institute for Polymer Research, Ackermannweg 10, 55128 Mainz, Germany; ‡ Department of Chemistry and Biochemistry, Boise State University, 2133 Cesar Chavez, Boise, 83725 Idaho, United States; § Multiphase Chemistry Department, Max Planck Institute for Chemistry, Hahn-Meitner-Weg 1, 55128 Mainz, Germany

## Abstract

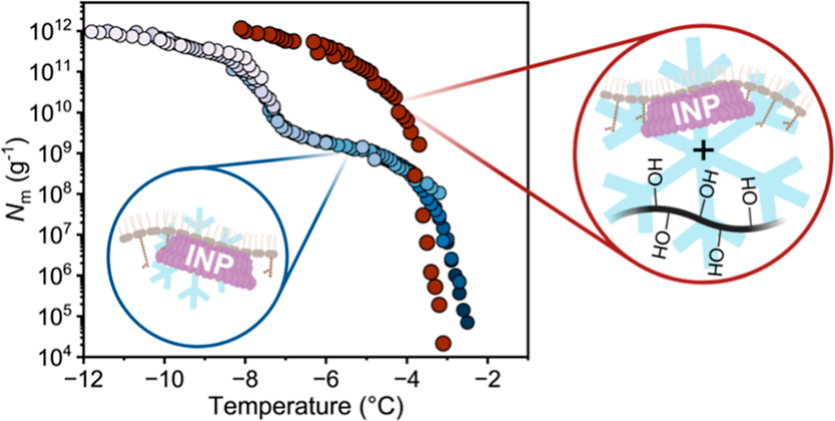

Ice-nucleating proteins (INPs) from bacteria like *Pseudomonas syringae* are among the most effective ice nucleators known. However, large INP aggregates with maximum ice nucleation activity (at approximately −2 °C) typically account for less than 1% of the overall ice nucleation activity in bacterial samples. This study demonstrates that polyols significantly enhance the assembly of INPs into large aggregates, dramatically improving bacterial ice nucleation efficiency. Simple compounds like polyvinyl alcohol increased the abundance of large INP aggregates by a factor of 100. This remarkable boost in ice nucleation efficiency is attributed to the stabilization of INP aggregates through membrane–polyol interactions that stabilize INP interactions and reduce structural fluctuations. The ability to regulate the abundance of large INP aggregates in bacterial ice nucleators enables fine-tuning ice nucleation processes at much lower concentrations for specific biomedical and technological purposes.

## Introduction

Ice nucleation is a critical process with far-reaching implications in various fields, from atmospheric sciences to biotechnology and food preservation. Precise control over ice nucleation is essential for optimizing processes, including cryopreservation of biological materials, food processing, and weather modification. However, achieving such control has remained challenging due to the complex nature of ice nucleation processes and the limited understanding of the molecular mechanisms involved. The ability to manipulate ice nucleation efficiently and predictably would not only advance our fundamental understanding of this phenomenon but also open up new possibilities for technological innovations. Therefore, developing methods to enhance and fine-tune ice nucleation activity is of paramount importance for both basic research and practical applications.

Among a wide variety of heterogeneous ice nucleators (INs) that facilitate ice formation by effectively overcoming the kinetic barriers of ice nucleation,^1−3^ the efficiency of INs from plant-associated bacteria such as *Pseudomonas syringae* is unmatched.^4^ The ability of these bacteria to catalyze freezing is attributed to ice-nucleating proteins (INPs) anchored to the bacterial outer membrane.^5^ They are a primary cause of frost damage in plants,^6^ and have been identified in atmospheric and precipitation samples, suggesting a role in cloud glaciation.^2,7^ A long-standing observation in the analysis of bacterial INs has been that the ice-nucleation active bacteria always display a spectrum of nucleation events with threshold temperatures ranging from −2 to −10 °C. Based on their activity, bacterial INs are usually classified in classes A to C, with threshold temperatures of −4.4 °C or warmer (class A), −4.8 to −5.7 °C (class B), and −7.6 °C and colder (class C).^8^ Pioneering studies have revealed that the differences in freezing temperatures are caused by INP assemblies of different sizes, with class A INs comprising the largest INP aggregates.^9,10^ These findings align with classical nucleation theory, which predicts that larger nucleation sites support higher ice nucleation temperatures.^11^

Recent studies have further explored the underlying size and distribution of INP aggregates in relation to experimentally observed freezing temperatures.^12,13^ Analysis of bacterial IN populations revealed that only 12% of all INs contribute to class A and only one IN per million cells is active at −2 °C.^13,14^ Class A INs have further been shown to require an intact cell membrane,^15,16^ and several studies have demonstrated that changing environmental conditions (e.g., pH, salts, temperature, cosolutes) mostly affect larger aggregates.^17−19^ Interestingly, improved efficiency of bacterial INs was recently observed in a well-balanced saline buffer, suggesting that the degree of aggregation can be manipulated by stabilizing INP aggregates and exerting beneficial conditions on protein–membrane interactions.^13^

Previous research has demonstrated that polyols can significantly influence the physical properties of lipid layers and stabilize proteins and protein–lipid interactions, allowing for precise control over molecular assembly and packing in specific applications.^20−22^ Building on this knowledge, we hypothesized that polyols could be strategically employed to manipulate INP aggregation, thereby offering a novel approach to fine-tune bacterial ice nucleation. To test this hypothesis, we systematically investigated the effects of common, biodegradable, water-soluble polyols, including glycerol, sorbitol, ethylene glycol (EG), and polyvinyl alcohol (PVA) on bacterial ice nucleation. Our results reveal that the carefully controlled addition of these polyols substantially enhances IN-activity, opening up new possibilities for their use in tailored freezing applications.

## Experimental Section

### Materials

Pure water was obtained from Millipore Milli-Q Integral 3 water purification system (Merck Chemicals GmbH, Darmstadt, Germany), autoclaved at 121 °C for 15 min, and filtered through a 0.1 μm bottle top filtration unit (VWR International GmbH, Darmstadt, Germany). Polyols were obtained from Alfa Aesar [PVA, (98–99% hydrolyzed, low molecular weight ∼17,600 to 26,400), Alginic acid sodium salt (low viscosity)]. Snomax was purchased from SMI Snow Makers AG (Thun, Switzerland) and consists of a preparation of inactivated bacteria cells of *P. syringae*. Dulbecco’s phosphate-buffered saline (DPBS) (without CaCl_2_ and MgCl_2_), MOPS and HEPES buffer were purchased from Sigma-Aldrich (Darmstadt, Germany).

### TINA Experiments

Ice nucleation experiments were performed using the high-throughput Twin-plate Ice Nucleation Assay, which has been described in detail elsewhere.^23^ In a typical experiment, a sample with a concentration of 1 mg/mL IN in a water/buffer–polyol mixture was prepared. This sample was serially diluted 10-fold by a liquid handling station (epMotion ep5073, Eppendorf, Hamburg, Germany). 96 droplets (3 μL) per dilution were placed on two 384-well plates and tested with a continuous cooling-rate of 1 °C/min from 0 to −30 °C with a temperature uncertainty of ±0.2 °C. The droplet-freezing was determined by two infrared cameras (Seek Thermal Compact XR, Seek Thermal Inc., Santa Barbara, CA, USA). The obtained fraction of frozen droplets was used to calculate the cumulative number of INs using the Vali formula.^24^ Experiments were performed multiple times with independent samples. Background freezing of pure water occurred at ∼−25 ± 2 °C.

### Folch Extraction

Folch extraction (FE) was performed by a protocol adapted from Wessel and Flugge.^25^

In short, *P. syringae* was dissolved in water at a concentration of 10 mg/mL. Five mL of the solution was pipetted in a 50 mL falcon tube, 20 mL methanol was added, and the mixture was vortexed thoroughly. Then, 10 mL chloroform was added, and the solution was vortexed again. After the addition of 15 mL water, the mixture was generously vortexed and centrifuged at 13,000*g* for 1 min. The resulting sample contained a large aqueous layer on top, a circular flake of protein in the interphase, and a smaller chloroform layer at the bottom. The upper layer was carefully removed, 15 mL methanol was added, vortexed, and centrifuged at 13,000*g* for 2 min. All samples were dried under vacuum.

### Ice Affinity Purification

Rotary ice-shell purification was used to purify the ice-nucleating biomolecules of Snomax. Details of the purification method have been described elsewhere.^26,27^ In short, ∼20 to 30 mL of water was used in a 500 mL flask to form an ice-shell using a dry ice-ethanol bath for 30–60 s. The flask was then rotated in a temperature-controlled EG bath, and the temperature of the bath was set to −2 °C. 50 mL precooled bacterial IN solution was added, and the flask rotated continuously in the bath until 30% of the solution was frozen. The obtained ice was melted and freeze-dried to obtain a mixture of present ice-binding proteins from *P. syringae*.

## Results and Discussion

**Figure 1:**
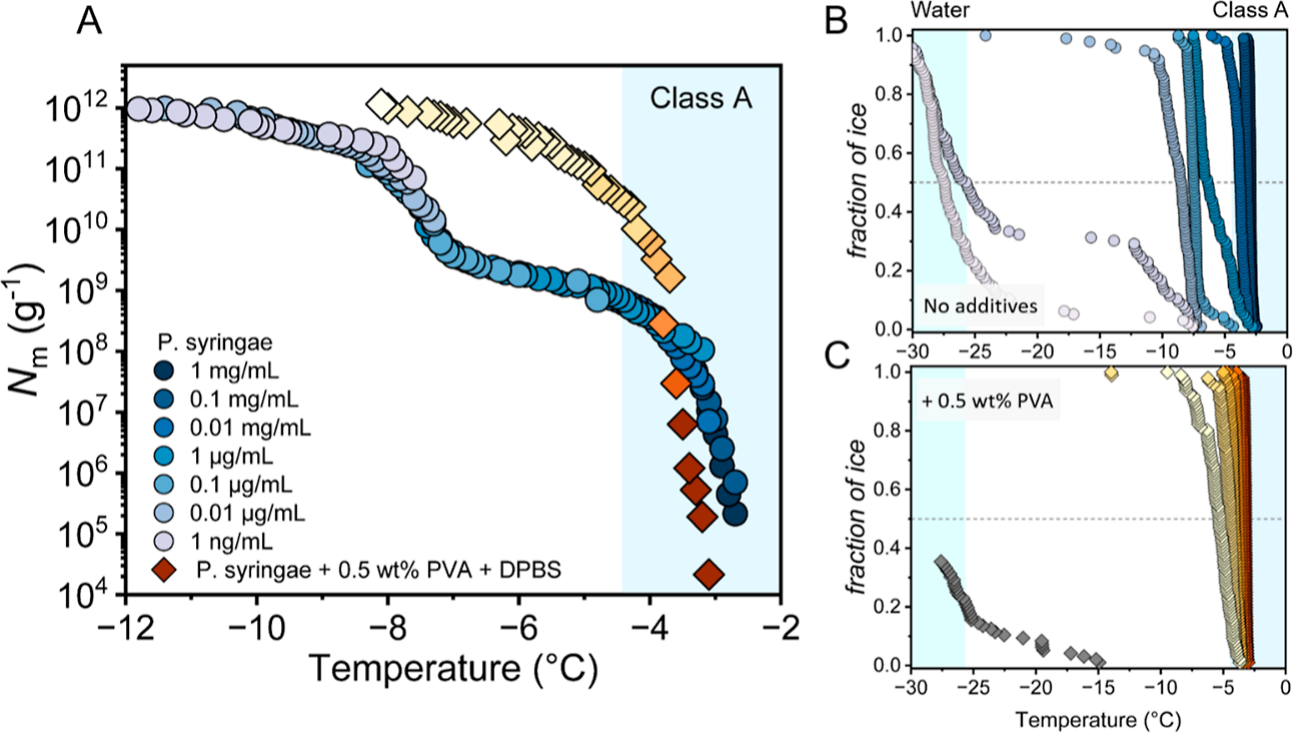
Freezing experiments of aqueous samples containing bacterial INs from *P. syringae* and in the presence of 0.5 wt % PVA in DPBS buffer. (A) Cumulative number of IN per unit mass of sample (*N*_m_) plotted against temperature. (B) Fraction of frozen droplets (*f*_ice_) for different *P. syringae* dilutions. Symbol colors indicate data from droplets with different concentrations and are identical to the plots shown in A. (C) *f*_ice_ for different *P. syringae* dilutions in the presence of 0.5 wt % PVA. Symbol colors represent different concentrations and are identical to concentrations used in (B). The dark gray data points represent a 0.5 wt % PVA control sample in DPBS buffer. The blue-shaded regions represent the temperature ranges for class A INs (>−4.4 °C) and when pure water freezes in our system (<−25 °C), respectively.

Figure 1A shows the results of freezing experiments of a dilution series of inactivated bacteria cells of *P. syringae* (Snomax) in water and a 0.1 M DPBS solution containing 0.5 wt % PVA. *P. syringae* solutions were serially diluted 10-fold from 1 mg/mL to 0.1 ng/mL, at constant DPBS and PVA concentration. The cumulative IN number (*N*_m_) was calculated using Vali’s formula and represents the number of active INs per unit weight above a certain temperature.^24^ The freezing spectra of the bacterial INs in water show two increases in the *N*_m_(*T*), at −2.9 and −7.5 °C with plateaus between −4.5 and −7 °C and below −9.5 °C. The two increases indicate that the IN-activity of *P. syringae* stems from two distinct IN classes with different activation temperatures. Plateaus occur when fewer INs are active at these temperatures. Based on the activation temperature, we assign the observed INs to class A and C, while class B INs are not identified in the cumulative freezing spectrum, consistent with previous works.^18,23,28,29^

The presence of 0.5 wt % PVA in buffered solution causes substantial changes in the freezing spectrum. The activity at ∼−7.5 °C is absent; instead, a single rise is observed, centered at −3.1 °C.

This indicates that PVA strongly promotes the formation of the more efficient class A INs. The overall number of INs remained constant, implying that no loss of INs occurred, and the presence of PVA in DPBS induced the formation of highly efficient class A INs. The small shift in the initial temperature of class A INs from −2.9 to −3.1 °C is due to the colligative freezing point depression. Importantly, PVA and other polyols do not show IN-activity by themselves in our measurements (Figure S1).

The drastic enhancement of aggregation upon PVA addition becomes even more apparent when droplet freezing statistics are used to compare the *T*_50_-values of the different solutions, as shown in Figure 1B. The *T*_50_-value is defined as the temperature at which 50% of the droplets are frozen. For *P. syringae* in water, the two main IN classes are clearly recognized at ∼−2.9 and ∼−7.5 °C. The third rise at ∼−25 °C corresponds to the freezing point of pure water in our system. We observe that the maximal IN-activity caused by class A INs (>−4.4 °C) for aqueous *P. syringae* solutions only occurred at very high concentrations (1 to 0.01 mg/mL). For the PVA-containing samples, we find that maximum IN-activity prevails to concentrations as low as 0.01 μg/mL, drastically improving the efficiency of *P. syringae*. We define the bacterial efficiency as the lowest concentration at which class A IN-activity at temperatures above −4.4 °C prevails. By this definition, the presence of PVA increases the efficiency by at least 100-fold.

**Figure 2:**
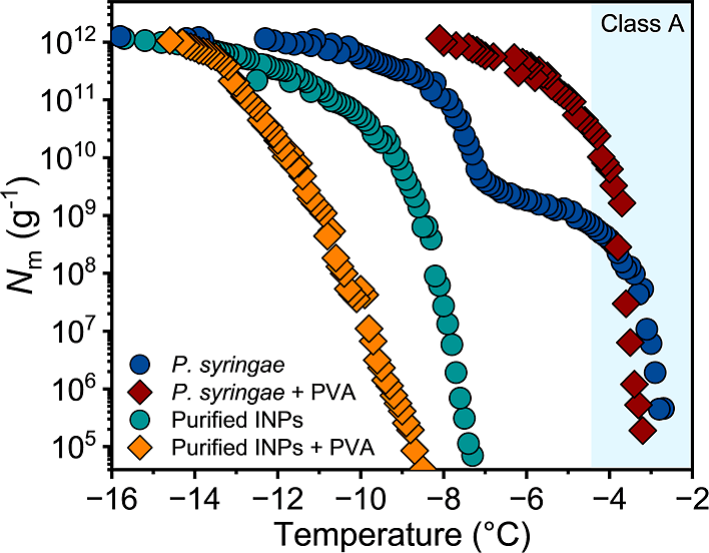
Freezing experiments of aqueous solutions of *P. syringae* (blue) and purified INPs (green) and in the presence of 0.5 wt % PVA (red, orange), all in DPBS buffer. *N*_m_ is plotted against temperature. The class A temperature range is shaded in blue.

Next, we explored whether the PVA enhancement effect results from direct polyol–protein interactions or arises from the facilitation of INP assembly through the stabilization of the membrane. To this end, we compared the IN-activity of PVA/*P. syringae* mixtures with PVA/purified INP mixtures in DPBS buffer. The first mixture retained the membrane to which the INPs are attached, whereas lipids and other macromolecules are largely removed from the second mixture. The INPs of *P. syringae* were purified using a combination of FE and ice affinity purification (IAP).^15^ FE separates lipid and protein components by partitioning lipids in a biphasic mixture of chloroform and methanol.^30^ IAP exploits the affinity of INPs to ice by incorporating them into a growing ice phase during the purification process while excluding impurities.^27^ Using this purification process, we obtained a mixture containing all INPs from the processed bacterial sample, including residual protein-associated lipids.^15^Figure 2 shows the freezing spectra of *P. syringae* and purified INPs in aqueous solutions as well as in the presence of PVA in DPBS buffer.

For the purified INPs, we observe an increase at ∼−7.5 °C, which is shifted to ∼−8.5 °C in the presence of PVA. Additionally, for both purified INP samples, the increase at ∼−2.9 °C is absent, and the total number of INPs is reduced. Clearly, the addition of PVA to the purified, membrane-free INPs did not result in an enhancement of activity as observed for the bacteria. This highlights the necessity of the intact membrane for class A formation and maximum IN-activity, and suggests that PVA stabilizes the INP-membrane system.^15,31^

**Figure 3:**
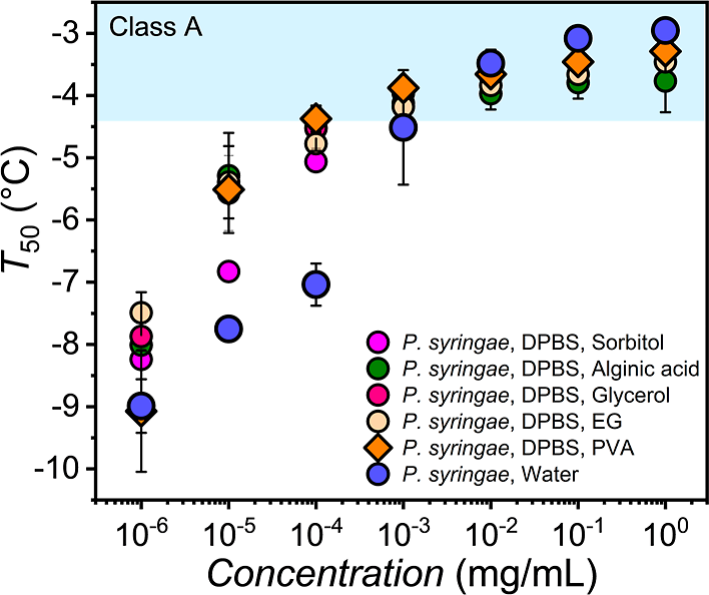
Freezing experiments of aqueous solutions of *P. syringae* in the presence of 1 wt % sorbitol (pink), glycerol (red), EG (beige), and in the presence of 0.5 wt % PVA (orange) and alginic acid (green), all in DPBS buffer. The *T*_50_-values are shown as a function of *P. syringae* concentration. Error bars represent the standard deviation of 2–5 independent measurements. The class A temperature range is shaded.

To investigate whether the enhancement of bacterial INs is a common phenomenon or a specific effect of PVA, different polyols were evaluated. Figure 3 shows *T*_50_-values of droplet freezing measurements for buffered solutions of *P. syringae* in the presence of different polyols, at 0.5 to 1 wt % concentration. The corresponding data is reported in the Supporting Information (Figure S2).

We find that the enhancement of freezing temperatures is primarily observed for *T*_50_-values below the class A region. For the highest *P. syringae* concentrations, the *T*_50_-values in buffer and in the presence of polyols are similar, which is expected since *P. syringae* shows the highest IN-activity at these concentrations. At lower *P. syringae* concentrations, we observe that all of the investigated polyols increase the *T*_50_-values. At the lowest concentrations, the polyols have little effect, presumably because the INP concentration is too low for the polyols to promote aggregation. Our findings of improved efficiency of bacterial ice nucleation in the presence of polyols disagree with previous reports showing that polyglycerol and PVA copolymers inhibit bacterial IN-activity.^32^ This discrepancy can be explained by different experimental conditions. All our measurements were performed in DPBS-buffered solutions since even slight changes in the solution pH are known to inhibit bacterial IN-activity.^17^ In fact, we find that adding PVA alone alters the pH of the solution and that bacterial INs in aqueous PVA solutions show reduced activities (Figure S3). This pH shift is likely caused by residual hydroxide and acetate groups present in PVA, which can explain the observed inhibition in previous reports. This highlights the necessity to ensure pH stability in PVA solutions.

**Figure 4:**
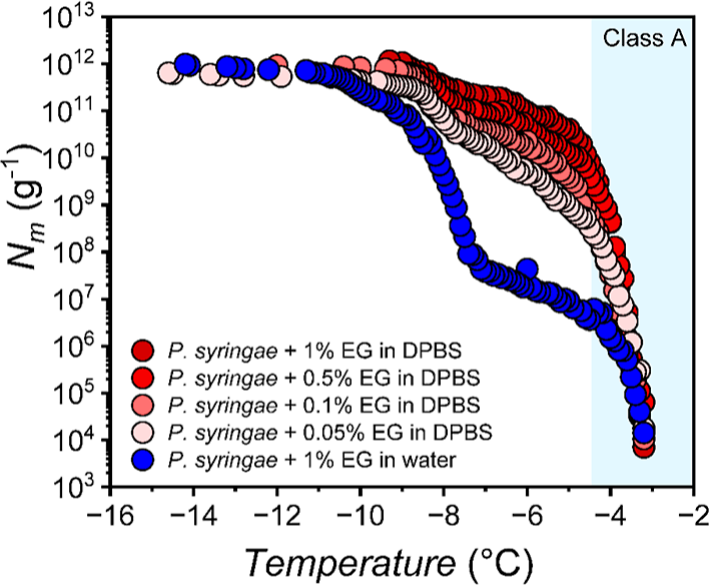
Freezing experiments of aqueous solutions of *P. syringae* in the presence of different amounts of EG in DPBS buffer. Symbol colors represent different EG concentrations, ranging from 0.05 wt % (light red) to 1 wt % (dark red). The addition of 1 wt % EG in aqueous solution of *P. syringae* (blue) was used as a control measurement. The class A temperature range is shaded.

While polyols enhance bacterial IN efficiency also in other buffers (Figure S4), their optimal performance is observed in DPBS. It has been previously demonstrated that DPBS is capable of enhancing INP aggregation and promoting the formation of class A INs.^13^ The enhancement effect was explained by limiting electrostatic interactions between the predominantly negative membrane and INPs, thus promoting interactions between INPs for aggregation. Polyols exhibit similar properties, which stabilize protein–protein and protein–lipid interactions.^20−22^ Indeed, adding polyols intensifies the stabilizing effects of DPBS on aggregation. Figure 4 shows the effect of different EG concentrations in DPBS on the freezing spectrum of *P. syringae*, ranging from 0.05 to 1 wt %. The formation of class A INs is observed to increase in DPBS with higher polyol concentration.

## Conclusion

Here we show that PVA, EG, and other polyols affect the formation of INP aggregates within the bacterial membrane, increasing bacterial ice nucleation efficiency at least 100-fold. Adding polyols to the bacteria further eliminated the known instability and inherent fluctuations in freezing temperatures.^19^ Polyols are known to stabilize liposomes and have been suggested to affect cell membrane properties.^33^ We hypothesize that the polyols form a protective adlayer on the membrane surface that stabilizes intramolecular INP interactions and reduces structural fluctuations, leading to precise arrangement of INPs to larger aggregates.^31,33^ The molecular details of the stabilization mechanism remain unknown but could involve changes in the viscoelastic properties of the membrane or perturbations of the lipid organization and local curvatures. This hypothesis would agree with experimental observations that class A INs are not expressed well in fluid membrane lipids.^16^ The ability of polyols to control the degree of bacterial INP aggregation is extraordinary. It paves the way for using bacterial INs in tunable freezing applications for biomedical and technological applications at much-reduced concentrations.

## Supporting Information Available

Ice nucleation measurements of polyol solutions in water and in buffer; ice nucleation measurements of *P. syringae* in aqueous PVA solution and in other buffers (PDF)
