# Supplementary material for: Polyol-Induced 100-Fold Enhancement of Bacterial Ice Nucleation Efficiency
Source: J Phys Chem C Nanomater Interfaces. 2024 Dec 5;128(50):21604–8. doi: 10.1021/acs.jpcc.4c07422 (PMC11664577; doi:10.1021/acs.jpcc.4c07422)
Supplement: Supplementary file 1 — jp4c07422_si_001.pdf [file jp4c07422_si_001.pdf]

Supporting Information:

Polyol-Induced 100-Fold Enhancement of Bacterial Ice Nucleation Efficiency

Authors: Galit Renzer[1], Rosemary J. Eufemio[2], Ralph Schwidetzky[1], Janine Fröhlich-Nowoisky[3], Mischa Bonn[1], and Konrad Meister*[1,2]

Affiliations:

[1]Department of Molecular Spectroscopy, Max Planck Institute for Polymer Research, 55128 Mainz, Germany

[2]Department of Chemistry and Biochemistry, Boise State University, 83725 Boise, ID, USA

[3]Multiphase Chemistry Department, Max Planck Institute for Chemistry, 55128 Mainz, Germany

Correspondence to: meisterk@mpip-mainz.mpg.de

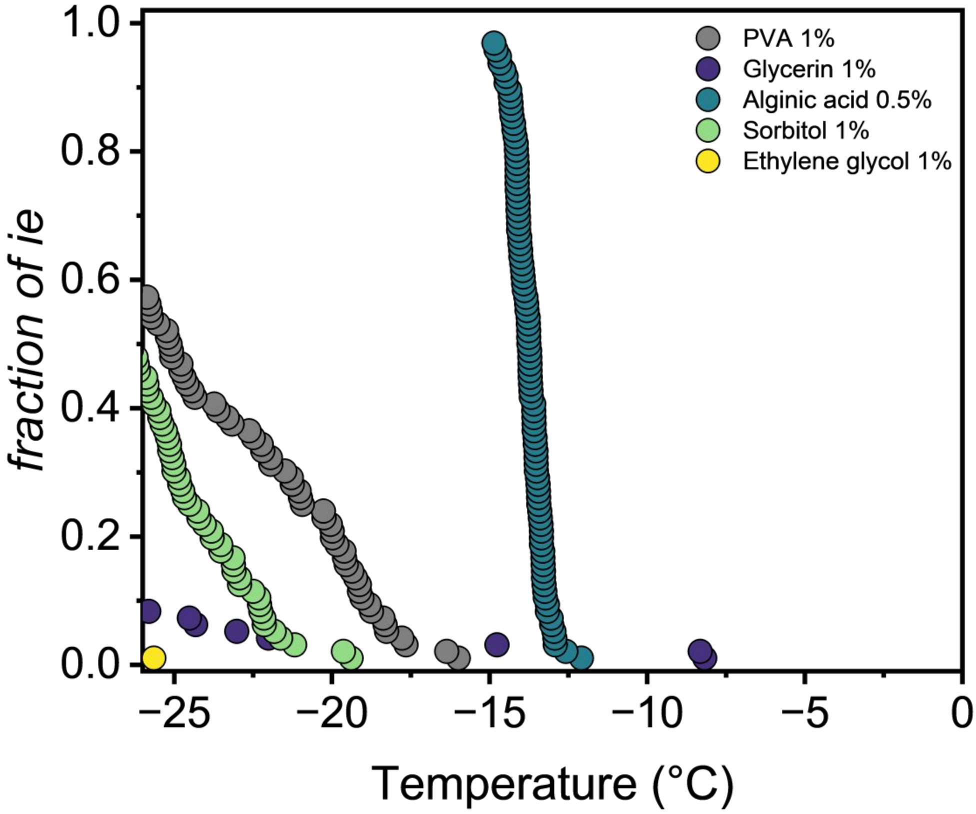

Figure S1: Freezing experiments with aqueous polyol solutions in DPBS buffer. Shown is the fraction of ice f_{ice} for different 0.5 and 1 wt% polyol solutions in DPBS buffer.

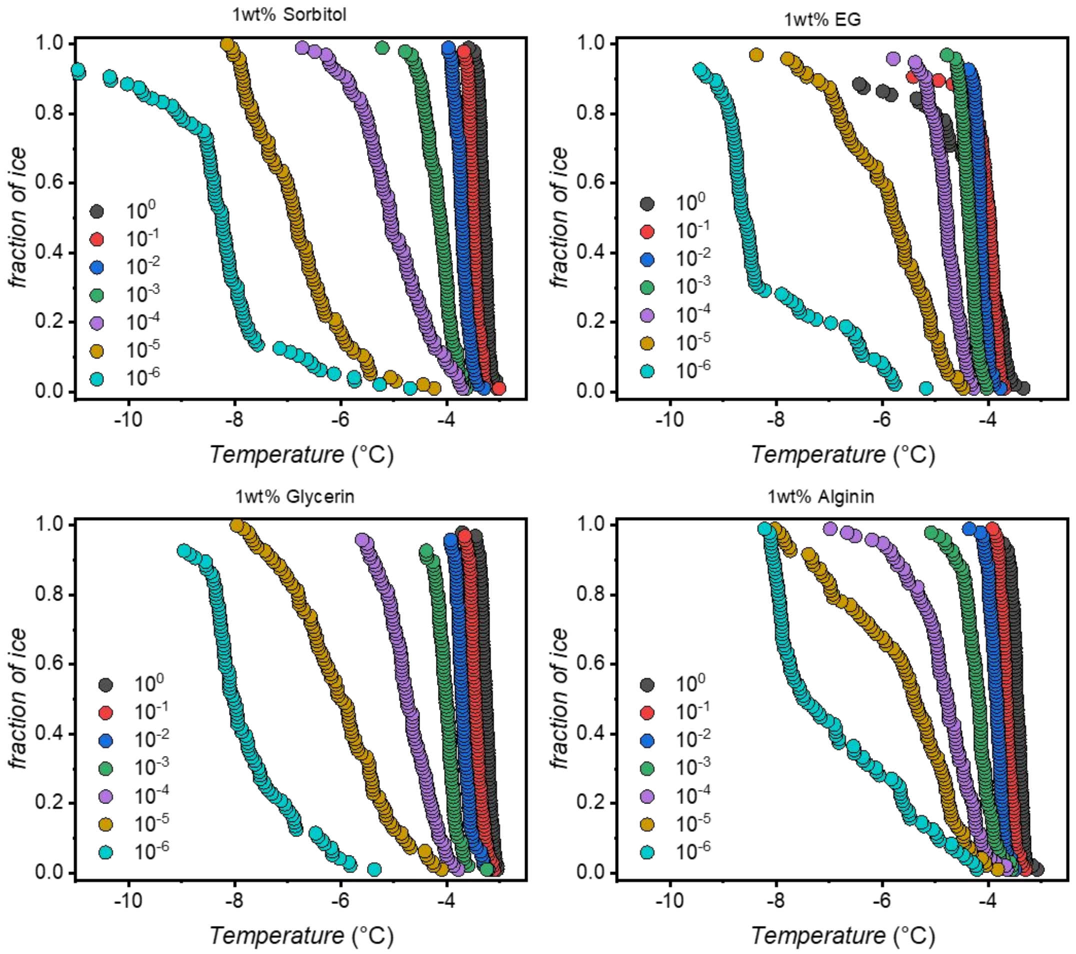

Figure S2: Freezing experiments with aqueous polyol solutions in DPBS buffer. Shown are the fraction of ice f_{ice} for different 1 wt% polyol solutions in DPBS buffer. Symbol colors represent different concentrations.

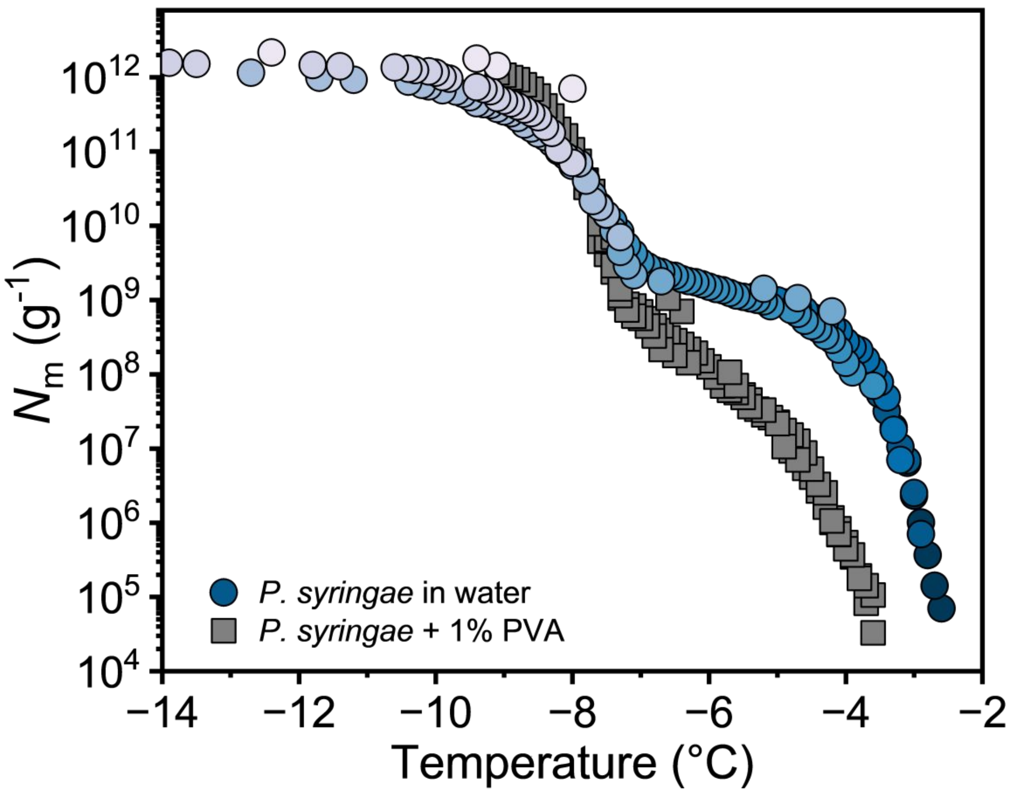

Figure S3: Freezing experiments with aqueous solutions of *P. syringae* in water and the presence of 1 wt% PVA. Shown is the cumulative number of ice nucleators per unit mass of sample (N_m) plotted against temperature.

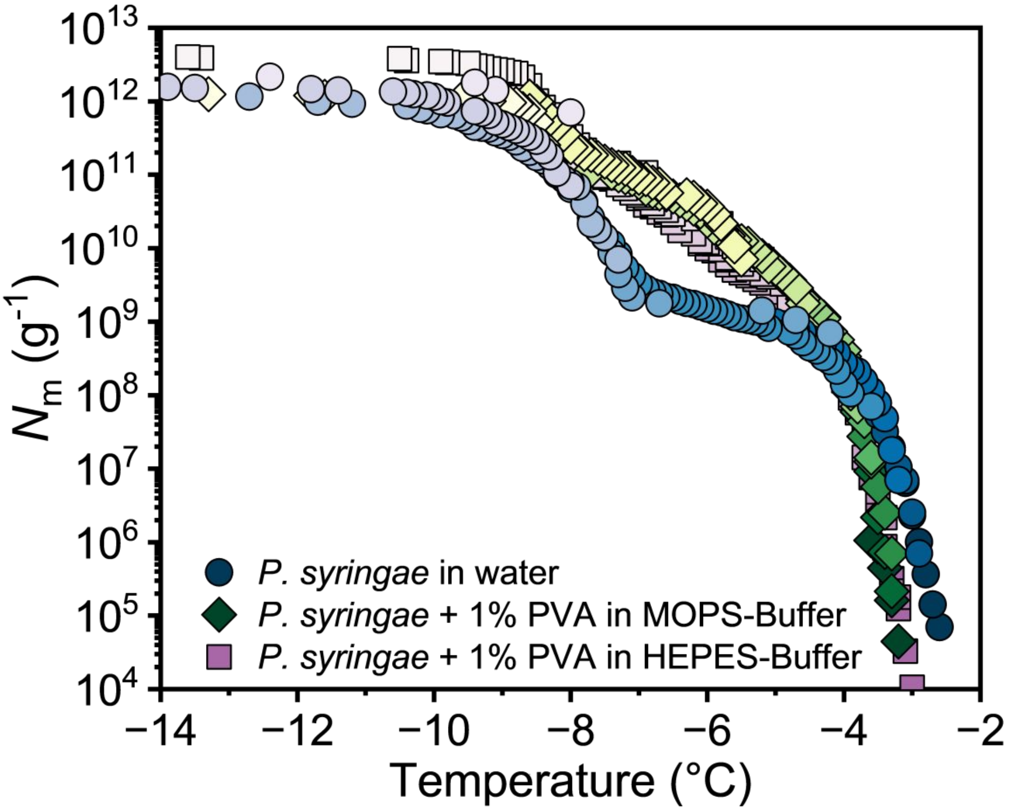

Figure S4: Freezing experiments with aqueous solutions of *P. syringae* in the presence of 1 wt% PVA in MOPS buffer (green) and HEPES buffer (purple). The freezing spectrum of *P.syringae* in water (blue) is shown as a control measurement. Symbol colors represent different concentrations. Shown is the cumulative number of ice nucleators per unit mass of sample (N_m) plotted against temperature.
